# Improving growth properties and phytochemical compounds of *Echinacea purpurea* (L.) medicinal plant using novel nitrogen slow release fertilizer under greenhouse conditions

**DOI:** 10.1038/s41598-020-70949-4

**Published:** 2020-08-14

**Authors:** Fatemeh Ahmadi, Abbas Samadi, Amir Rahimi

**Affiliations:** 1 Department of Soil Science, Faculty of Agriculture, Urmia University, Urmia, Iran; 2 Department of Plant Production and Genetics, Faculty of Agriculture, Urmia University, Urmia, Iran

**Keywords:** Plant sciences, Environmental sciences

## Abstract

Medicinal plant production is most important than other agricultural plants due to their phytochemical compounds effects on human health. Paying attention to plant nutrition requirement is so important. In order to assess the effect of nitrate (NO_3_^−^) dosage supplies from two types of fertilizers on growth and phytochemical properties of *Echinacea purpurea* rhizomata cum radicibus, an experiment with completely simple design was carried out under greenhouse conditions. Two types of fertilizers (new invented nitrogen (N) slow release fertilizer and urea chemical fertilizer) at three dosages (50, 100, and 150 mM) were applied. Plant growth parameters and total phenolic (TPC), total flavonoids (TFC), polysaccarides content, essential oil content, caffeic acid derivatives, and anti-radical scavenging activities of *E. purpurea* were assessed. The results showed the significant (*p* ≤ 0.01) differences among treatments, both in growth and phytochemical properties. Using of N slow release, especially in 150 mM dosage, significantly increased all the plant growth and phytochemical properties. The dried *E. purpurea* rhizomata cum radicibus contained more caftaric acid (max 12.56 mg g^−1^ DW) and chicoric acid (max 7.56 mg g^−1^ DW) than other derivatives. Despite the impact of heavy metals on yield and growth of *E. purpurea*, the concentration of all heavy metals and micronutrients (boron (B), cadmium (Cd), copper (Cu), iron (Fe), manganese (Mn), molybdenum (Mo), nickel (Ni), lead (Pb), and zinc (Zn)) in studied soil and fertilizer samples was less than United States Environmental Protection Agency (USEPA) limits of contamination. Based on the results, using of N slow release fertilizers can improve phytochemical properties of the plant due to its polymeric structure and can be a suitable substitution of chemical fertilizers, especially in medicinal plants growth.

## Introduction

Many of researchers are pay attention to herbal and medicinal plants as natural remedies due to their bioactive compounds recently^1,2^. *Echinacea purpurea* (L.) Moench is one of the most important medical plants of *Asteraceae* family with various pharmacological effects^3^. The major three species of *Echinacea* are known as *Echinacea angustifolia*, *Echinacea purpurea*, and *Echinacea pallida*. *E. purpurea* is native to much of the United States and its morphological properties are also known as purple coneflower with orange and cone-shaped flowering head that change to purple, rose, or white petals during June to September^4^. It has a narrow leaves and three feet height stem with dark, thick and pungent rhizomata cum radicibus^5^. In the environment, *E. purpurea* grows randomically along water canals with low densities for affective harvest for commercial purposes^6^.

Comparing of other *Echinacea* species, *E. purpurea* is the most able species to adjust to new conditions. It is resistance to heat or cold weather conditions, easy to grow, and flaunt high efficiency^7,8^. Numerous scientific researches demonstrated the useful effect of *Echinacea* immunomodulatory functions and human health^9,10^. The doctors explored that the alcohol extract of *Echinacea* rhizomata cum radicibus immediately increased phagocytic activity and leads to high production of white blood cells, as well as, various in vitro researches has confirmed the phagocytosis, and antibody-dependent cellular cytotoxicity effects of *E. purpurea* rhizomata cum radicibus extracts^4^. Decreasing of severity and symptoms of the common cold by using *E. purpurea* extracts was reported in several clinical studies^11^. According to immunological studies, *E. purpurea* enhanced activation of the nonspecific cellular and increased humoral immunity by increasing the activation and production of monocytes, lymphocytes, leukocytes and cytokines^12^. Based on the studies, the immune‐stimulatory property is the most important property of the species that involves the immune system based on the dose level^13^. Although the rhizomata cum radicibus of *E. purpurea* is very beneficial, purple coneflower of the species was known to be useful for the improvement of the various illnesses, such as sore throats, coughs, and snake bite^8^. Meanwhile, *E. purpurea* has been used to treat viral, bacterial and fungal infections^14^ and the healing of the burns and wounds^15^.

Various chemical components of *E. purpurea* were associated with its biological activities and medicinal effects^3,16^. For instance, the main phenolic compounds of *E. purpurea* were found as caffeic acid esters and chicoric acid, as well as, polysaccarides was dominant in *E. purpurea* rhizomata cum radicibus extracts^17^. Recent studies demonstrated that the polysaccharide content leads to enhance the macrophage activity and the cytokine production^18^. Also, the antifungal and antiviral properties of *E. purpurea* were related to certain groups of the phenolic compounds and alkamides^18–20^. Scavenging of the free radicals and lipid peroxidation assay were related to the antioxidant properties of leaves and rhizomata cum radicibus extracts of *E. purpurea*^21^. Higher concentration of isobutyl amide was affects the *Echinacea*’s anti‐inflammatory property^17^.

Overall, good manufacturing of high quality medicinal plants is depend on precise aware of plant nutrient requirement. The study in Hungary demonstrated that application of 60–80 kg ha^−1^ N, 40–60 kg ha^−1^ phosphorus (P) and 80–100 kg ha^−1^ potassium (K^+^) lead to increase of biological yield and phenolic compounds of *E. purpurea*^22^. The optimum nutrients ratio for plant growth depends on species and environmental conditions^23^ and soil pollution with chemical pollutants such as various heavy metals^24^. Several studies demonstrated that soil heavy metals pollution caused to decrease of *E. purpurea* yield under greenhouse condition^14^. Among all nutrients, N, due to cooperation in phytochemical compound’s structures had very critical effect on *Echinacea* yield and medicinal properties^13^. So, attention to N supply has key role in chemical and medicinal composition of *Echinacea*. Previous researches illustrated that the various plant growth parameters of *E. purpurea* are generally improved with optimum mixture ratio of NO_3_^−^ and ammonium (NH_4_^+^) in comparison with either N form alone^16,17^. The NO_3_^−^ dosage can affect not only rhizomata cum radicibus morphological properties, but also the overall rhizomata cum radicibus biomass^25^. The results of Verma et al. (2019) showed that phenolic compounds (caftaric acid, cholorogenic acid, cynarin, echinacoside and chicoric acid) were significantly affected by NO_3_^−^ dosage.

Urea is most popular conventional fertilizers in agriculture which is partially absorbed by plants. This results in reduce N usage efficiency for crops and environmental pollution^26^. In recent years, slow release fertilizer has been developed. Slow release fertilizer releases nutrients according to plants requirement for physiological functions during the long time, results in increase of fertilizer efficiency^27^.

Although there are many previous reports about growing medicinal plants in different culture media and NO_3_^−^ dosages, there is no prior research on comparison of phytochemical properties of *E. purpurea* growing in the presence of novel invited N slow release fertilizer and common chemical fertilizer. So the goal of this research was to compare the growth properties and phytochemical compounds of *E. purpurea* growing with various amounts of NO_3_^−^ dosage supplied from slow release and urea fertilizers.

## Materials and methods

### Experimental background

The experiment was carried out in a greenhouse complex at Urmia University, West of Azarbaijan Province, Iran. Seeds of *E. purpurea* obtained from Pakan Bazr Esfahan, an Iranian private joint stock company, on April. 15, 2019. The seeds were put into a mixture of perlite and peat moss substrates for initial growth. Irrigation operation was performed regularly as needed depending on greenhouse condition. Seedlings were harvested after one month when they were at 3–4 true leaves stage and transplanted in the experimental pots which had one plant grown in a pot (2.5 L) containing a mixture of soil (2-mm sieved) and fine sand for better aeration and leaching, under greenhouse conditions. The density of production system was chosen according to Waidyanatha et al. (2020). The set point for the greenhouse temperature and humidity were 19–21 °C (night–day) and 75% respectively. Fertigation was performed based on *E. purpurea* nutrition need, shown in Table 1.

**Table 1:** Nutrition requirement of *E. purpurea* (Seif Sahandi et al.^21^).

Nutrient	Attribute (mM)
Nitrogen (N)	150.0
Phosphorus (P)	2.0
Potassium (K)	6.2
Calcium (Ca)	3.9
Magnesium (Mg)	2.0
Sulfur (S)	4.2
Iron (Fe)	50.0
Manganese (Mn)	9.0
Zinc (Zn)	0.8
Copper (Cu)	0.8
Boron (B)	18.0
Molybdenum (Mo)	0.5
pH	6.5–7.5
EC (dS m^−1^)	< 2

**Figure 1:**
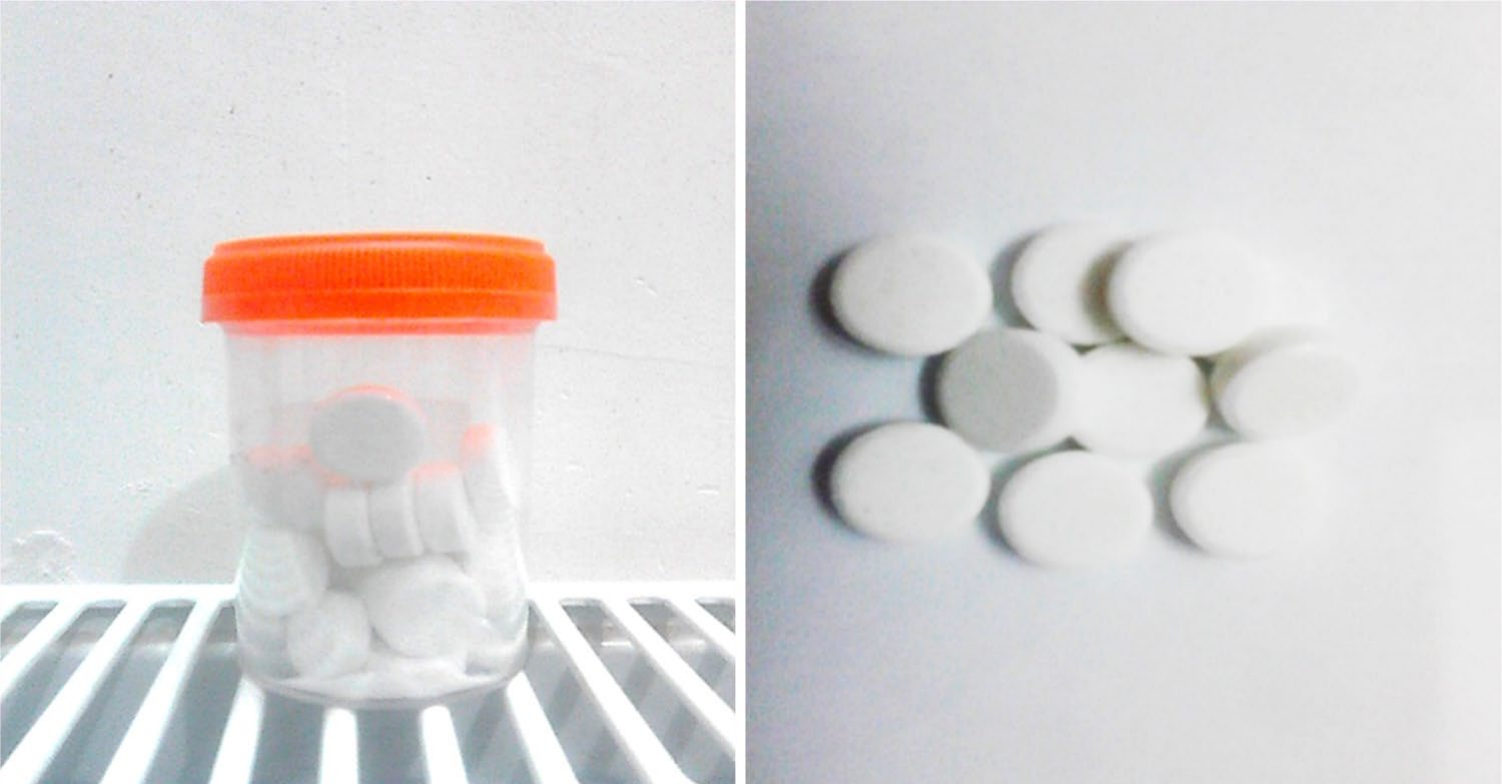
Nitrogen slow release fertilizer.

All essential elements except N were supplied from the soil adequately, but two types of N fertilizers were used for supply of enough N nutrition need including slow release N fertilizer, and Urea. Chemical composition of used various fertilizers were analysed according to standard methods^28,29^. The amount of N application was determined based on the differences of plant N requirement and soil N, according to fertilizer type. In this study 50 (less than adequate), 100 (moderate), and 150 (adequate) mM N were provided from each fertilizer types. Slow release fertilizer used in this study, was invited based on a novel composition at science and technology center of Hamedan province, Iran and registered at Iranian Patent Office, Tehran on Jan. 30, 2017 (patent number 139550140003013815). Details of the novel N fertilizer and its Figure were shown in Table 2 and Fig. 1 respectively.

**Table 2:** Main properties of invited slow release fertilizer.

Properties	Details
Physical shape	Compact and tablet form
Weight (g)	2.3
Diameter (cm)	1.5
Height (cm)	0.6
Harness and solubility	6.4/10
Adhesion strength (%)	100
Surface area (mg g^−1^)	200
Colour	White
Main composition	Powdered cotton seed, Semi-solid sucrose, and water
Heavy metals	None

The fertilizer can control the N release during the plant grow period based on complex formation between the chelator agent and the element. The main difference that distinguish this novel fertilizer from usual and commercial slow release fertilizers is its chelator agent that is more efficient and environmental friendly. As well as, urea fertilizer was chosen as common N fertilizer among Iranian farmers. All experimental treatments were repeated as three replicates with less than 1% standard deviation. Plants were fertigated for 240 days, every 10 days, under control conditions in greenhouse. The rhizome with (rhizomata cum radicibus) of plants were harvested after growing period and dried at 23–25 °C (at room temperature), then phytochemical analyses were performed.

### Soil analysis

Soil chemical and physical properties were measured based on standard methods. Soil pH and electrical conductivity (EC) were measured using 1:5 w/v soil to water ratio suspension^11^. Organic matter (OM) was determined after oxidation of soil organic fraction with potassium dichromate (K_2_Cr_2_O_7_) and sulfuric acid (H_2_SO_4_)^30^. Cation exchange capacity (CEC) was determined with 1 mol L^−1^ sodium acetate solution (pH 8.2) and 96% ethanol^14^. Calcium carbonate (CaCO_3_) was measured after boiling of 2.5 g soil with 25 mL of 0.5 N hydrochloric acid (HCl)^12^. Three fractions (sand, silt, and clay) of soil particles were determined following the pipette method^15^.

Soil calcium (Ca^2+^) and magnesium (Mg^2+^) were measured titrimetrically, K^+^ was determined by flame photometry, sulfate (SO_4_^2−^) by spectrophotometric and turbidimetric methods, NO_3_^−^ and phosphate (PO_4_^3−^) by colorimetry with an ultraviolet–visible (UV–Vis) spectrophotometer^11^. Available micronutrients (Cu, Fe, Mn, Mo, and Zn) and heavy metals (Cd, Ni, Pb) were extracted with a solution containing 0.005 mol L^−1^ diethylene triamine penta acetic acid (DTPA), 0.01 mol L^−1^ calcium chloride, and 0.1 mol L^−1^ (pH 7.3) triethanolamine (TEA), as well as, boron (B) concentration was determined based on hot water method^26,31^. The concentration of micronutrients were measured by Atomic Absorption Spectrophotometer (AAS) (Model Varian Spectra-220).

### Fertilizer analysis

The urea and sloe release fertilizer were passed through a 0.5-mm sieve after air-dried at 25 ± 1 °C. Total N, NH_4_^+^ and NO_3_^−^ concentrations in samples were determined according to Kjeldahl method^27^. Heavy metals and trace elements (Cd, Cu, Fe, Mn, Ni, Pb and Zn) content in samples were determined after digestion with aqua regia based on standard method^18,32^.

### Plant growth parameters

After harvesting the plants, different growth parameters such as plant height (cm) and total fresh leave weight (g plant^−1^), fresh rhizomata cum radicibus weight (g plant^−1^), chlorophylls a and b (mg g^−1^ FW), and leaf area (cm^2^) were measured. Weighted 0.5 g of dry sample was homogenized with 10 mL acetone^23^. Homogenized samples were centrifuged at 10,000 rpm for 15 min. The supernatant was separated and the absorbance spectra were measured at 400–700 nm. Maximum absorbance of chlorophylls a and b were measured in 662 nm and 645 nm, respectively^29^. So that^6,10^,

1 $$ {\text{Chlorophyll a}} = { 11}.{\text{75 A}}_{{{662}}} {-}{ 2}.{35}0{\text{ A}}_{{{645}}} $$

2 $$ {\text{Chlorophyll b }} = { 18}.{\text{61 A}}_{{{645}}} {-}{ 3}.{96}0{\text{ A}}_{{{662}}} $$

### Essential oil content

Essential oils were extracted from 75 g dry weight of rhizomata cum radicibus of the plant^17^. The samples were oven-dried at 25 °C for 3–5 days. Rhizomata cum radicibus samples was mixed with 1,000 mL deionized water in a 2-L round-bottomed flask and the essential oil was extracted based on hydrodistillation method by clevenger trap^17^.

### Polysaccharide

The standard method was followed for determine of rhizomata cum radicibus polysaccharides. The 0.6 mL of sugar solution was mixed with the 0.3 mL phenolic solution in screw cap, capped and vortex-stirred tubes. 1.5 mL of H_2_SO_4_ (1 M) was added slowly down the side of the tube. The tubes were incubated for 30 min at room temperature (20 °C)^11,26^. The absorbances was determined at 490 nm using distilled water as blank in a UV–Vis spectrophotometer^26,33^.

### Total phenolic content

Dry and powder rhizomata cum radicibus was analysed for caftaric acid, cholorogenic acid, chicoric acid, cynarin and echinacoside based on standard method^13,30^. As, 40 mg of sample was transferred into a 15-mL centrifuge vial. The five bio active compounds were extracted with 4 mL of 70% methanol for 15 min using an ultrasonic sonicator^13^. The vials were centrifuged at 3,000 rpm for 10 min^30^. After filtration of supernatant by 0.2 μm nylon syringe filter, 300 μL of the filtered extract from each sample were transferred into a glass auto-sampler high performance liquid chromatography (HPLC) vial^13,34^. The HPLC system consisting of a controller, auto-injector, and a column oven. Samples were injected onto a Phenomenex Luna Qg column (5.0 μm; 4.6 × 150 mm) with a C_18_ guard column (4 × 3 mm)^30^. The mobile phase was 0.1% phosphoric acid (A) and acetonitrile (B) at a constant flow rate of 1.3 mL min^−1^^13^. The standard concentrations of 5, 25, 50, 100, and 200 μg mL^−1^ was used for standard curves plotting as a function of peak area in HPLC chromatograms^13^. The quantifications of caftaric acid, cholorogenic acid, chicoric acid, cynarin, and echinacoside were performed on the basis of the peak area of UV absorption at 330 nm with comparison to the standard curves of the authentic sample^13^.

### Total flavonoids content

The analysis of total flavonoid content in rhizomata cum radicibus extracts was performed by colorimetric method^35^. Briefly, 30 μL of the extract was added to 150 μL of sodium nitrate (5% W/V) followed by the addition of 3 mL of aluminum chloride hexahydrate (10% W/V) and incubated for 5 min^35^. The 1 mL of NaOH (1.0 M) was added to the mixture and diluted to the mark with distilled water. After incubation at room temperature in dark place for 30 min, the absorbance of the solution was measured at 415 nm by spectrophotometer. For the quantification of TFC, the quercetin (QE) was used as an external standard^35^.

### Anti-radical scavenging activity

The anti-radical scavenging activity of samples was evaluated based on the colorimetric method^12^. Briefly, 15 μL of methanolic extract was added to 2.0 mL of the 2, 2-diphenyl-1-picrylhydrazyl (DPPH) solution and the mixture was incubated in dark place at 20 °C for 30 min^12^. Then the absorbance was measured at 517 nm. The following equation was used to calculation of DPPH inhibition^12^:

3 $$ {\text{Inhibition }}\left( \% \right) \, = \, [({\text{A}}_{{{\text{control}}}} - {\text{A}}_{{{\text{sample}}}} ) \, /{\text{A}}_{{{\text{control}}}} ] \, \times {1}00 $$

where A_control_ and A_sample_ are the absorbance of the control and the sample respectively.

### Super oxide anti-radical scavenging activity

Super oxide anti-radical scavenging activity of samples was determined according to standard method^23,29^. As, 9 mL of 5 mM HCl buffer (pH 8.2) was mixed with 1 mL of the extract. Then, 40 μL of 4.5 mM pyrogallol was added to the mixture. The mixture was shaken for 3 min and the absorbance of the solution was measured at 420 nm by spectrophotometer^23^. The percentage of scavenging effect was expressed as^2^:

4 $$ {\text{Super oxide radical scavenging }}\left( \% \right) \, = \, \left[ {\left( {{\text{A}}_{0} - {\text{A}}_{{1}} /{\text{A}}_{0} } \right)} \right] \, \times {1}00 $$

where A_0_ is the HCl buffer absorbance and A_1_is the extract absorbance.

### Nitric oxide anti-radical scavenging activity

Nitric oxide anti- radical inhibition can be estimated by using Griess Illosvoy reaction according to standard method^19^. Briefly, 3 mL of 10 mM sodium nitroprusside and 0.5 mL of phosphate buffer saline solution was incubated at 25 °C for 150 min^19^. Then, 0.5 mL of the solution mixed with 1 mL of sulfanilic acid reagent and left for 5 min for completing diazotization. A pink coloured chromophore is formed after addition of 1 mL of naphthyl ethylene diamine dihydrochloride in diffused light^19^. The absorbance of these solutions were measured at 540 nm against. The nitric oxide radical inhabitation was expressed as following equation^14^:

5 $$ {\text{Nitric oxide radical inhibition }}\left( \% \right) \, = \, [({\text{A}}_{{{\text{control}}}} - {\text{A}}_{{{\text{sample}}}} ) \, /{\text{A}}_{{{\text{control}}}} ] \times {1}00 $$

where A_control_ and A_sample_ are the control and the extract absorbance respectively.

### 2,2′-azino-bis 3-ethylbenzothiazoline-6-sulfonic acid (ABTS) test

ABTS test is also a spectrophotometric method which is carried out using an improved ABTS decolourisation assay^18^. It is applicable for both lipophilic and hydrophilic compounds. ABTS^+^ was generated by oxidation of ABTS with potassium persulfate^18^. Three milliliter of ABTS cation solution were added to 30 mL methanol extract solution in 1 cm path length disposable micro cuvette and the decrease of absorption was measured during 6 min^18^.

### Statistical analysis

The statistical scheme was a completely simple design, including six treatments (50, 100, 150 mM slow release fertilizer and 50, 100, 150 mM urea) with three replications. Differences among means of treatments were analysed by Duncan’s multiple comparison at *p* ≤ 0.01. Statistical analysis was performed using statistical analysis software (SAS). All determinations were carried out in triplicate.

## Results

Main physiochemical properties of soil sample was given in Table 3. According to results, the soil had neutral reaction without salinity effect (EC less than 2 dS m^−1^) which is suitable for wide range of nutrients availability^20^. As well as, it had calcareous nature (more than 5% CaCO_3_ at topsoil) with sandy clay loam texture. Soil organic matter was at moderate level (1–1%) in agricultural calcareous soils.

**Table 3:** Physiochemical properties of studied soil.

	pH	EC	OM	CEC	CaCO_3_	Sand	Silt	Clay	Soil texture
		(dS m^−1^)	(%)	(cmol_c_ kg^−1^)	(%)	(%)			
Soil sample	7.20	1.03	1.12	17.51	12.26	63	16	21	Sandy clay loam

Macro and micro nutrient concentrations in soil solution were shown in Tables 4 and 5.

**Table 4:** Soluble anions and cations concentration in studied soil extract (1:20 soil to distilled water ratio).

	Ca^2+^	Mg^2+^	K^+^	SO_4_^2−^	PO_4_^3−^	NH_4_^+^	NO_3_^−^
	(meq L^−1^)						
Soil sample	5.50	3.32	2.51	3.78	4.93	0.73	2.45

**Table 5:** Available concentration and total content of trace elements in studied soil sample.

	B	Cd	Cu	Fe	Mn	Mo	Ni	Pb	Zn
	(mg kg^−1^)								
Available	0.65	0.10	5.04	102.50	81.57	0.15	4.36	1.09	10.72
Total	1.19	0.21	46.52	2,300.54	520.63	1.68	20.68	8.36	43.65

The results relevant that the different heavy metals content varied depending on the element and fertilizer type. Among heavy metals, Zn had the highest content (maximum 3.1 mg kg^−1^), whereas Cd showed the lowest (maximum 0.21 mg kg^−1^) in two types of N fertilizers. The results were in accordance with previous studies^13,36^. Iron generally does not cause toxicity to plants. However, a high concentration of Fe can decrease P plant availability due to the formation of iron-phosphate salts and it is therefore harmful to plants indirectly^24^. The content of various heavy metals in all N-fertilizer types were as fallowed: Fe > Mn > Zn > Ni > Cu > Pb > Cd. Among these heavy metals, Cu, Mn, Zn, and Fe are considered as nutritionally essential elements, Ni is classified as an element with possible beneficial health effects^22^, and Cd and Pb are regarded as elements with potentially toxic and environmentally hazardous effects^19^. Slow release fertilizer contains more essential element contents than urea (Table 6). The knowledge of trace element and heavy metals content in different chemical fertilizers is useful for estimation of the environmental potential risks resulting from high loading application of fertilizers^8^. In Table 7, the background mean content of trace elements and heavy metals in worldwide soils are presented^21^.

**Table 6:** Content of trace elements in different types of fertilizers.

Fertilizer	Cd	Cu	Ni	Pb	Zn	NO_3_^−^
	(mg kg^−1^)					(%)
Urea	0.21	1.5	2.6	0.5	3.1	46
Slow release	0.01	2.1	3.8	0.06	5.4	32

**Table 7:** Mean background contents of trace elements in surface soils (Khalaf et al.^12^).

Heavy metals	Background content in soils
	(mg kg^−1^)
Cd	0.41
Cu	38.90
Fe	45,000
Mn	488
Ni	29
Pb	27
Zn	70

**Figure 2:**
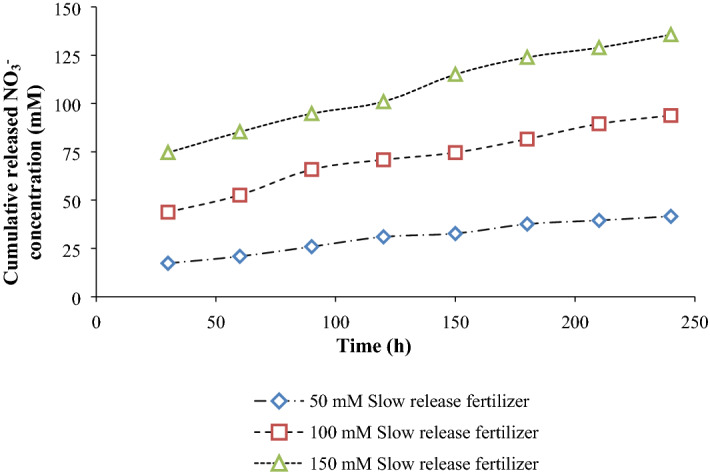
Kinetics of NO_3_^−^ release from treated soil with slow release fertilizer.

Cumulative release of NO_3_^−^ from slow release fertilizer at different concentrations during the time is shown in Fig. 2. Passing the time up to 200 h resulted in an increasing release of NO_3_^−^ from slow release fertilizer. Increasing the NO_3_^−^ concentration leads to increase of release rate over time. The highest (130 mM) amount of NO_3_^−^ released was observed in 150 mM slow release fertilizer (Fig. 2). However, NO_3_^−^ from the urea chemical fertilizer showed a maximum release (125 mM) within 1 to 2 h (data not shown).

Plant grow parameters of *E. purpurea* under different two types of fertilizers were shown in Table 8. Increasing of NO_3_^−^ dosage caused to increase in plant height and rhizomata cum radicibus weight significantly (Table 8). All plant growth parameters were significantly more in slow release fertilizer treatments than urea.

**Table 8:** Some morphological properties of *E. purpurea* growing under different fertilizer treatments.

Property	Slow release fertilizer			Urea		
	50 mM	100 mM	150 mM	50 mM	100 mM	150 mM
Height (cm)	74.60^c^ ± 2.53	83.10^b^ ± 2.82	105.20^a^ ± 3.21	49.30^e^ ± 2.10	66.80^d^ ± 2.25	71.50^c^ ± 2.60
Total fresh leave weight (g plant^−1^)	21.86^bc^ ± 0.28	25.67^b^ ± 0.56	33.19^a^ ± 1.13	14.22^d^ ± 0.11	19.23^c^ ± 0.15	23.14^b^ ± 0.49
Fresh rhizomata cum radicibus weight (g plant^−1^)	41.29^c^ ± 1.15	53.20^b^ ± 1.22	65.43^a^ ± 1.40	16.32^e^ ± 0.75	24.10^d^ ± 0.90	29.36^d^ ± 0.98
Chlorophyll a (mg g^−1^ FW)	12.3^6c^ ± 1.23	15.24^b^ ± 1.32	20.10^a^ ± 1.56	9.89^e^ ± 0.62	12.10^c^ ± 0.84	16.35^d^ ± 0.55
Chlorophyll b (mg g^−1^ FW)	4.13^bc^ ± 0.14	5.21^b^ ± 0.16	7.54^a^ ± 0.26	3.10^d^ ± 0.11	4.56^bc^ ± 0.14	5.19^b^ ± 0.19
Leaf area (cm^2^)	38^c^ ± 1.26	43^b^ ± 2.26	55^a^ ± 2.96	22^e^ ± 0.59	30^d^ ± 0.91	36^c^ ± 1.52

Data were expressed as the mean of three replicates ± standard deviation (n = 3).

Values within a row followed by different letters are significantly different at the 0.01 probability level.

The effects of slow release and chemical fertilizers on total phenolic and flavonoid contents, polysaccarides and essential oil content of *E. purpurea* rhizomata cum radicibus are shown in Table 9. The significant differences were obtained among phytochemical properties in slow release and chemical fertilizers treatments. The highest total phenol was recorded in the 150 mM slow release fertilizer treatment. Increasing of NO_3_^−^ concentration in slow release fertilizer significantly leads to increase of total phenolics, flavonoids, polysaccarides, and essential oil content.

**Table 9:** Phytochemical analysis of *E. purpurea* rhizomata cum radicibus under various treatments.

	Slow release fertilizer			Urea		
	50 mM	100 mM	150 mM	50 mM	100 mM	150 mM
Total phenolic compound (GAE g^−1^ DW)	47.3^c^ ± 0.31	51.6^b^ ± 0.30	60.2^a^ ± 0.56	12.3^f^ ± 0.52	30.6^e^ ± 0.60	43.5^d^ ± 0.15
Total flavonoid content (QE g^−1^ DW)	19.2^cd^ ± 0.37	24.9^b^ ± 0.48	32.8^a^ ± 0.21	10.5^e^ ± 0.17	14.6^de^ ± 0.31	22.6^bc^ ± 0.20
Polysaccarides (mg g^−1^ DW)	27.6^c^ ± 0.42	38.4^b^ ± 0.11	50.5^a^ ± 0.75	13.9^d^ ± 0.53	28.6^c^ ± 0.18	36.9^b^ ± 0.47
Essential oil content (% w w^−1^)	0.09^de^ ± 0.001	0.18^c^ ± 0.01	0.29^a^ ± 0.01	0.06^e^ ± 0.001	0.13^d^ ± 0.01	0.24^b^ ± 0.01

Data were expressed as the mean of three replicates ± standard deviation (n = 3).

Values within a row followed by different letters are significantly different at the 0.01 probability level.

**Figure 3:**
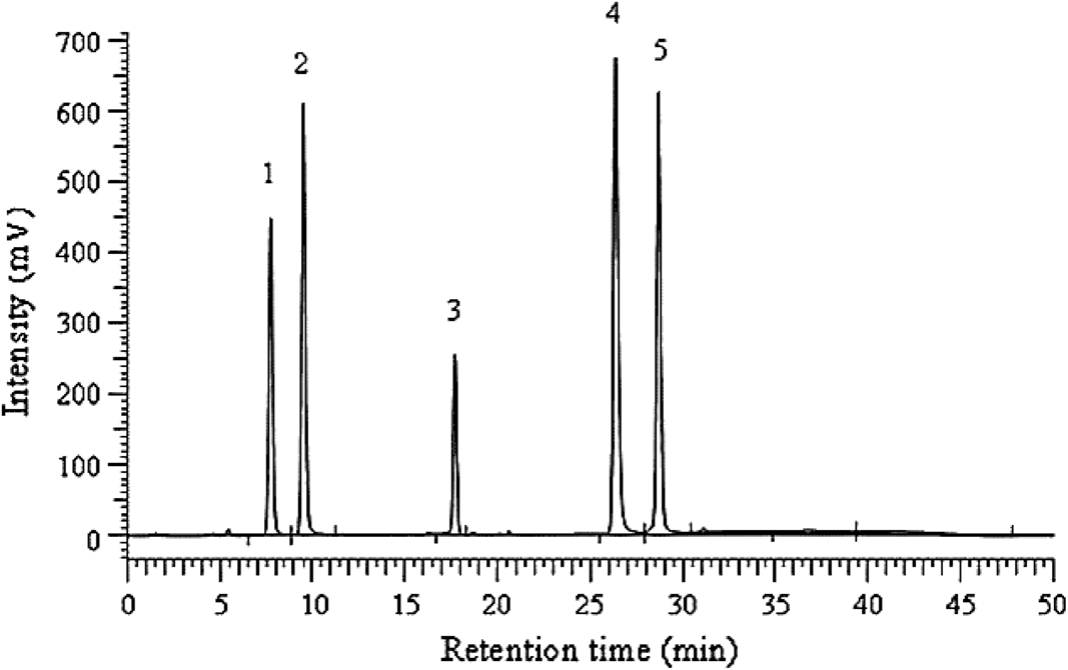
HPLC profile of a standard mixture of caffeic acid derivatives. Peak 1, caftaric acid; 2, chlorogenic acid; 3, echinacoside; 4, chicoric acid; and 5, cynarin.

The HPLC profile of a standard mixture of caffeic acid derivatives is shown in Fig. 3. Results of caffeic acid derivatives content as affected by slow release and chemical fertilizers in dry rhizomata cum radicibus of *E. purpurea* are shown in Table 10. Caffeic acid derivatives content was affected by fertilizer types and dosages. The dried *E. purpurea* rhizomata cum radicibus contained more cichoric acid and caftaric acid than other derivatives (Table 10). These results are in agreement with previous reports^18,19^.

**Table 10:** Caffeic acid derivatives of *E. purpurea* rhizomata cum radicibus under various treatments.

Caffeic acid derivatives	Slow release fertilizer			Urea		
	50 mM	100 mM	150 mM	50 mM	100 mM	150 mM
Caftaric acid (mg g^−1^ DW)	2.31^d^ ± 0.11	7.13^b^ ± 0.15	12.26^a^ ± 0.02	0.78^e^ ± 0.02	1.06^de^ ± 0.03	3.83^c^ ± 0.06
Chlorogenic acid (mg g^−1^ DW)	0.23^cd^ ± 0.07	0.95^b^ ± 0.02	1.36^a^ ± 0.02	0.16^e^ ± 0.01	0.43^d^ ± 0.01	0.73^c^ ± 0.01
Echinacoside acid (mg g^−1^ DW)	0.89^bc^ ± 0.01	1.32^b^ ± 0.06	2.66^a^ ± 0.01	0.29^d^ ± 0.01	0.75^cd^ ± 0.01	0.96^bc^ ± 0.01
Chicoric acid (mg g^−1^ DW)	1.23^e^ ± 0.06	4.56^c^ ± 0.08	7.65^a^ ± 0.65	0.74^f^ ± 0.01	2.03^d^ ± 0.04	5.44^b^ ± 0.09
Cynarin (mg g^−1^ DW)	0.68^c^ ± 0.01	2.88^b^ ± 0.03	5.26^a^ ± 0.04	0.43^c^ ± 0.01	0.98^c^ ± 0.02	3.28^b^ ± 0.05

Data were expressed as the mean of three replicates ± standard deviation (n = 3).

Values within a row followed by different letters are significantly different at the 0.01 probability level.

The effects of various different fertilizers on anti-radical scavenging activity of *E. purpurea* rhizomata cum radicibus were shown in Table 11. The nitric oxide, DPPH, super oxide and ABTS anti-radicals were assesses in the present study.

**Table 11:** Different anti-radical scavenging activity of *E. purpurea* rhizomata cum radicibus under various treatments.

Anti-radical	Slow release fertilizer			Urea		
	50 mM	100 mM	150 mM	50 mM	100 mM	150 mM
DPPH (%)	25.36^b^ ± 0.95	28.95^ab^ ± 0.36	31.22^a^ ± 0.33	12.62^d^ ± 0.36	20.31^c^ ± 0.18	26.12^b^ ± 0.26
Super oxide (%)	15.39^cd^ ± 0.73	19.21^bc^ ± 0.55	23.66^b^ ± 0.65	12.36^d^ ± 0.98	13.65^d^ ± 0.42	28.76^a^ ± 0.14
Nitric oxide (%)	28.24^bc^ ± 0.68	32.19^b^ ± 0.48	40.36^a^ ± 0.15	19.21^d^ ± 0.23	26.25^c^ ± 0.36	31.42^b^ ± 0.36
ABTS (%)	10.23^d^ ± 0.59	15.23^b^ ± 0.67	20.95^a^ ± 0.29	8.26^d^ ± 0.51	12.84^c^ ± 0.11	16.63^b^ ± 0.47

Data were expressed as the mean of three replicates ± standard deviation (n = 3).

Values within a row followed by different letters are significantly different at the 0.01 probability level.

Although there are characterized by excellent reproducibility, but all of them were different in their response to antioxidants under certain conditions. The highest nitric oxide (40.36%), DPPH (31.22%), super oxide (23.66%), and ABTS (20.95%) anti-radical scavenging activities were found in the 150 mM slow release fertilizer treatment, which could be due to higher phenolic content, whereas the lowest various anti-radical scavenging activities were obtained in the 50 mM urea treatment.

## Discussion

Previous researches showed that soil CEC was varied as affected by organic matter content^4^. In fact, increasing of soil CEC results from the increasing of negative charge surface sites on soil colloids due to decomposition of organic matter in pH value above 3 (point of zero charge (PZC) of organic matter)^30^. Increasing of soil CEC leads to increase of nutrients retention at active soil exchangeable sites which plays critical role in nutrition release and their availability for plant^15^.

In comparison with *E. purpurea* nutrition requirement (Table 1), most of essential nutrients (except NO_3_^−^) in soil solution were in at adequate range which can supply plant essential macro and micro nutrition requirement without adding any fertilizer during the time. However, NO_3_^−^ concentration in soil solution was noticeably (more than 3 times) less than *E. purpurea* requirement that indicates the need to NO_3_^−^ fertilizers apply in order to increase NO_3_^−^ level in the studied soil.

Nitrate leaching from agricultural soils is common due to anionic nature of NO_3_^−^ ion and non-adsorption on negatively charged soil exchangeable sites, which leads to apply of different N fertilizers to soil^7^. In present study, the soil N deficiency was calculated with regard to *E. purpurea* N-requirement and provided the optimum level of NO_3_^−^concentration for the plant growth by using different N fertilizers containing various percentages of NO_3_^−^. Numerous research have reported the harmful influence of soil pollution and heavy metals concentration on *E. purpurea* growth and medicinal properties^32^. According to Table 5, the studied soil contains different heavy metals available concentration less than (more than 4 times) dangerous limit in agricultural soils^30^, which reported the 0.2, 13.0, 6.0, 16.0, and 22.0 mg kg^−1^ as acceptable limit for available Cd, Cu, Ni, Pb, and Zn in soil respectively.

The data show the lower total concentration of heavy metals in studied soil sample than USEPA limits, which reported the 1.5, 200, 50, 200, and 400 mg kg^−1^ for Cd, Cu, Ni, Pb, and Zn respectively. So, in the present study, it can be mention that there is no risk of heavy metal pollution for *E. purpurea* growth. The application of mineral fertilizers which have contaminants of trace elements may impose concern regarding the entry and toxic accumulation of these elements in agro ecosystems^37^. So, the chemical composition of 3 types of N fertilizers was analyzed for their content of NO_3_^−^, HN_4_^+^, and heavy metals (Table 6).

By comparing these values with the contents of trace elements in different N fertilizer types (Table 6), it is apparent that the content of all heavy metals in used N fertilizers in present study are lower than their natural background in soils. Therefore, in soils receiving these fertilizers, heavy metals cannot be enriched. So, in present study, the main source of these trace elements are the soil and only the soil can supply these elements for the plant growth mainly. Inspite of micro nutrients, N content of different studied fertilizers was shown in Table 6. Based on obtained results, urea (46% N) had higher NO_3_^−^ content followed by slow release fertilizer (32%). Different NO_3_^−^ fertilizers varied significantly in their N content that can affect the plant growth. However, NH_4_^+^ content in all N fertilizers changed in narrow range, so at the relatively constant NH_4_^+^ content in the soil, the soil NO_3_^−^ content affects the plant phytochemical properties.

The presence of polymeric compounds used in the slow release fertilizer structure leads to the gradual maintenance and release of NO_3_^−^ from the fertilizer and these compounds are not present in the urea fertilizer. Gradual release of NO_3_^−^ from slow release fertilizers results in degradation of NO_3_^−^ availability for the plant and prevention of leaching and loss of soil.

It is well known that the elasticity of the cell wall plays an important role in cell division^6^. The osmotic pressure in the cell increase by increasing of NO_3_^−^ concentration, leading to sufficient swelling pressure for cell division that affect the morphological properties^14^. Meanwhile, the NO_3_^−^ dosage can influence growing substrate pH, especially in nutrient recirculating systems^18^. It has been found that an increase of NO_3_^−^ dosage caused a significant increase of medicinal and phytochemical compounds of *E. purpurea* due to improve of rhizomata cum radicibus growth^31^. Similar results were found by Sidhiq et al. (2020) in *E. purpurea* plants grown in floating raft system for 21 weeks. Nitrogen is a predominant element in chlorophyll structure. Increasing of NO_3_^−^ dosage significantly affects the chlorophylls content^21^. Increasing of chlorophyll content caused to increase of leaf area due to increase of light adsorption^36^. The results were in consistent with Attarzadeh et al. (2019) who found that higher NO_3_^−^ dosage resulted in higher leaf area, rhizomata cum radicibus and total biomass.

According to Saeed et al. (2018), accumulation of phenolic compounds in the plant rhizomata cum radicibus prevents NO_3_^−^ toxicity in plants. There are some reports showing that stress conditions increase either phenylalanine ammonia lyase (PAL) synthesis or activity in plants^6,11,12,14,32^; other authors^24^ have found that some stress treatments delay the increase in wound-induced PAL activity. Phenylalanine Ammonia-Lyase activity was found to vary greatly with the stage of plant development^18^. The results were in accommodation with Zaushintsena et al. (2019). Variation between and among the herb species also exists due to geographical location, stage of development, time of harvest, and growth conditions^1^.

Caffeic acid derivatives are sensitive to NO_3_^−^ dosage, and increasing NO_3_^−^ concentration will result in a significant increase of caffeic acid derivatives in *E. purpurea* (Table 10). Results showed high caffeic acid derivatives content with slow release fertilizers than urea. It may be due to slow release of NO_3_^−^ during the time and more availability for plants.

Increasing of NO_3_^−^ dosage in slow release fertilizer leads to significant increase of all anti-radical scavenging activities. Rhizomata cum radicibus extract of *E. purpurea* can entrap hydroxyl radicals. This antioxidant mechanism is described as elimination of free radicals and chelating metal ions^38^. Effective compounds of *E. purpurea* are alkylamids, polysacharides, glicoproteins and chicoric acid. Chicoric acid is the main phenolic compound with antioxidant effect in *E. purpurea*. Phenolic compounds like caffeic acid and chlorogenic acid are the most efficient naturally occurring antioxidants. As the result of the presence of chicoric acid in leaf extraction of *E. purpurea*, this species has the most antioxidant effect among all other^4^. Beside chicoric acid, typical constituents of *E. purpurea* extracts are echinacoside, chlorogenic acid, cynarin and caftaric acid. All of them are able affect the free radical production and lipid peroxidation^6^. Recent studies also relevant that echinacoside and caffeic acid derivative have weak anti-bacterial and antiviral effects and are protectants against reactive oxygen species^20,39^. The antioxidant activity could be ascribed to the polyphenolic components^13^, such as flavonoids^34^, phenolic acids^35^ or phenolic diterpenes^9^.

It was found that the concentration of all heavy metals in soil samples and fertilizers studied was less than the USEPA limits of contamination. With this point of view, there were significant differences in morphological and phytochemical properties of *E. purpurea* with respect to slow release and chemical N fertilizers containing different dosages of NO_3_^−^. All morphological and phytochemical properties were higher in the presence of slow release fertilizer than urea chemical fertilizer. It may be due to the polymeric structure of slow release fertilizer that control the N release during the time. Increasing of morphological and phytochemical properties, caffeic acid derivations and anti-radical scavenging activities were observed by increasing of NO_3_^−^ dosage. With regard to future growth conditions of the plant, it is important to make operational additional research projects for medicinal plants and flowers grown under greenhouse conditions, with the objective of building further confidence in the advantages of the plant cultivation and importance of NO_3_^−^ concentration control in environmental systems.
